# Coffee arabica research (1932–2023): Performance, thematic evolution and mapping, global landscape, and emerging trends

**DOI:** 10.1016/j.heliyon.2024.e36137

**Published:** 2024-08-10

**Authors:** Siddig Ibrahim Abdelwahab, Manal Mohamed Elhassan Taha, Ahmed Ali Jerah, Ieman A. Aljahdali, Bassem Oraibi, Hassan Ahmad Alfaifi, Saleh M. Abdullah, Amal Hamdan Alzahrani, Omar Oraibi, Yasir Babiker, Abdullah Farasani

**Affiliations:** a Health Research Centre, Jazan University, Jazan, Saudi Arabia; b Department of Medical Laboratory Technology, Faculty of Applied Medical Sciences, Jazan University, Jazan, Saudi Arabia; c Department of Clinical Laboratory Sciences, Taif university, Taif, Saudi Arabia; d Pharmaceutical Care Administration (Jeddah Second Health Cluster), Ministry of Health, Saudi Arabia; e Department of Pharmacology and Toxicology, College of Pharmacy, King Abdulaziz University, Saudi Arabia; f Department of Internal Medicine, Faculty of Medicine, Jazan University, Jazan, Saudi Arabia; g Department of Surgery, Faculty of Medicine, Jazan University, Jazan, Saudi Arabia

**Keywords:** *Coffea arabica* L., Thematic evolution, VOSviewer, Bibliometrix, International collaboration, Research landscape and trends

## Abstract

**Background:**

Research on Coffea arabica focuses on various aspects, including genetics, breeding, climate change resilience, pest and disease management, agronomy, sensory analysis, and sustainability. This study aims to analyze the hotspots, conceptual map and dynamicity, global landscape, and emerging trends in *Coffea arabica* research (CA-R).

**Methods:**

A comprehensive dataset comprising data-driven articles (N = 3967) from 1932 to 2023 was extracted from Scopus using predefined search terms. VOSviewer and Bibliometrix applications were utilized to analyze the data. Thematic evolution was examined by identifying shifts in research focus over time. The global landscape was assessed by examining comparative productivity and collaborative dynamics. Highly-cited CA-R was identified to highlight key findings in specific research areas.

**Results:**

The analysis revealed a steady growth of CA-R (annual growth rate = 6.53 %), with strong international collaboration (international co-authorships = 29.35 %) and significant contributions from various countries. Brazil leads the way with 1601 publications, accounting for 28.55 % of the total. Recognizable CA-R focused on important areas such as pollination, shade management, nanotechnology applications, roasting effects, disease management, and environmental impacts. Thematic analysis identified five distinct clusters representing different CA-R themes: “coffee”, “coffea,” “fermentation,” “*Coffea arabica*,” and “climate change.” Emerging themes such as "*in vitro* culture," "sustainable agriculture," "climate change," and "coffee berry borer" were also identified.

**Conclusion:**

The current findings enhance our understanding of CA-R and lay the groundwork for future studies in the coffee industry.

## 1 Introduction

*Coffea arabica* L. (CA) is highly valued for its superior quality and flavor profile. High-quality Arabica beans are often associated with specific regions renowned for coffee production, such as Ethiopia, Saudi Arabia, Colombia, Brazil, Costa Rica, and Jamaica [1]. CA is cultivated in diverse regions worldwide, each contributing to the unique characteristics of the beans produced. Growing conditions, including altitude, climate, soil composition, and cultivation practices, greatly influence the flavor and aroma of coffee [2,3]. CA cultivation requires specific conditions to thrive. It is typically grown at higher altitudes ranging from 600 to 2000 m (2000 to 6500 feet) above sea level. The cooler temperatures combined with ample rainfall and well-drained soil create an ideal environment for CA plants. Shading provided by taller trees and a diverse ecosystem promotes healthy growth and enhances flavor development in coffee cherries [[3], [4], [5], [6], [7], [8]].

CA plants take several years to mature and produce fruit. The flowering and fruiting cycles are highly sensitive to environmental factors, and beans require careful harvesting and processing to maintain their quality. Hand picking is the preferred method for harvesting CA to ensure that only ripe cherries are selected, as the beans within each cherry ripen at different rates. This meticulous approach to harvesting contributes to the higher cost of CA compared with robusta [[9], [10], [11]]. Once harvested, coffee cherries undergo processing to separate the beans from the fruit. There are two primary methods of processing: the dry method (natural process) and the wet method (washed process). The dry method involves drying the cherries, whereas the wet method involves removing the fruit pulp before drying the beans. Each method imparts distinct characteristics to the final coffee cups [12,13].

The popularity and demand of CA have made it a significant economic commodity worldwide. The coffee industry provides livelihoods for millions of people, ranging from small-scale farmers to exporters, roasters, and baristas. Specialty coffee, which often features Arabica beans, has gained popularity in recent years, with consumers seeking unique flavor profiles and traceability in their coffee choices [1,14,15].

However, CA is not a challenge. Plants are susceptible to pests and diseases, such as leaf rust and berry disease, which can significantly affect yield and quality. Climate change poses an additional threat because rising temperatures, unpredictable weather patterns, and increased pest pressure can disrupt coffee production. Sustainable cultivation practices and ongoing research and development efforts are crucial to mitigate these risks and ensure the long-term viability of CA production [12,13,[16], [17], [18]].

Research on CA (CA-R) focuses on various aspects, including genetics [[19], [20], [21]], breeding [21], climate change resilience [22], pest and disease management [12], agronomy [5], sensory analysis [18,22], and sustainability [1,4,14]. CA-R contributes to enhancing coffee quality, productivity, and resilience while supporting the livelihoods of farmers and preserving the unique characteristics of CA. Bibliometric analysis is crucial in this study as it provides a systematic approach to evaluating the existing literature on CA-R. It uncovers citation patterns, publication trends, and thematic evolution, identifying research gaps and areas of high productivity. It assesses the impact and visibility of CA-R, reveals emerging trends, and offers a global perspective, fostering international collaboration. Overall, bibliometric analysis guides future research in CA-R by understanding the research landscape and informing decision-making [[23], [24], [25], [26], [27]]. Despite the significance of CA-R in the coffee industry, dedicated bibliometric assessments are lacking, presenting an opportunity for researchers. In this study, our primary research question is: "What is the current state of CA-R (1932–2023) and what are the key trends, impacts, contributors, and collaborations within the field?" By addressing this research question, we aim to gain a comprehensive understanding of the research landscape and its impact, providing valuable insights for further development and advancement in CA-R.

## 2 Materials and methods

### 2.1 Selection of scopus database

Scopus, a multidisciplinary database, encompasses various fields including health, life sciences, medicine, pharmacology, psychology, and nursing. It serves as an extensive resource for scholarly articles, making it highly suitable for researching diverse topics. What sets Scopus apart from other databases is its coverage of multiple subject areas and provision of comprehensive citation information. By indexing numerous journals and conference proceedings, Scopus proves particularly valuable for interdisciplinary research [28]. The data search and extraction for this study were conducted on May 1, 2024.

### 2.2 Search strategy and term generation

**Fig. 1:**
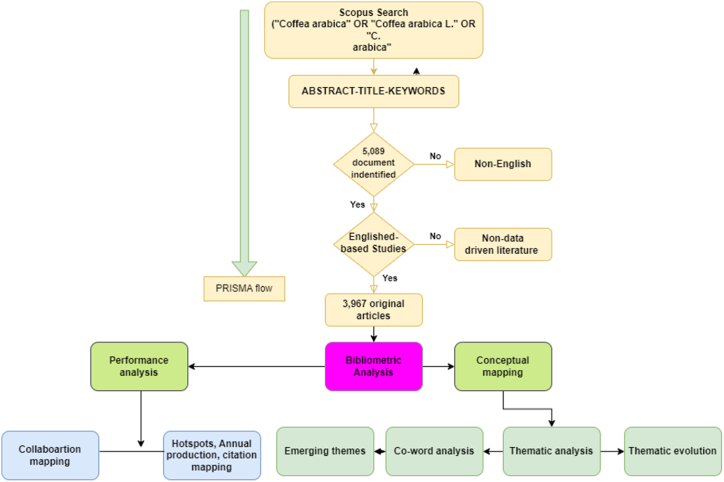
Search strategy in the Scopus database and the study design. The time span was 1932–2023 (n = 3967). This study used a bibliometric analysis and thematic evaluation based on the PRISMA guidelines.

The search strategy and term generation were conducted following a systematic step-by-step approach [29]. The strategy was initially tested in the Scopus database and adjusted to ensure relevant results were retrieved. The entire search process was thoroughly documented, including details such as the database used, search terms employed, and Boolean operators utilized. The search strategy was refined iteratively based on insights gained from the literature review. Fig. 1 provides a summary of all the steps undertaken in this study. Initially, the research question or topic of interest was clearly defined. Key concepts and main components of the research question were identified, and standardized terms were sought from the Kew Science Website [16]. Kew Science encompasses various botanical disciplines, including taxonomy, ecology, conservation, and genetics. The extracted terms ("*Coffea arabica*" OR "*Coffea arabica* L." OR "*C. arabica*") were combined using appropriate Boolean operators to construct search strings. The search strategy also incorporated specific inclusion/exclusion criteria, such as language or publication type. Truncation or wildcards were employed to capture variations of the search terms.

### 2.3 Selection of data-driven studies

The initial search yielded a total of 5089 documents, comprising 4666 articles (91.7 %), 153 reviews (3.0 %), 129 conference papers (2.5 %), 72 book chapters (1.4 %), 30 notes (0.6 %), 9 short surveys (0.2 %), 8 errata (0.2 %), 6 conference reviews (0.1 %), 4 books (0.1 %), 4 editorials (0.1 %), 4 letters (0.1 %), and 4 data papers (0.1 %). The findings reveal diverse literature sources, encompassing primary research articles and various other relevant publications. However, this study's focus was explicitly on original studies, leading to the exclusion of different document types. The inclusion criteria involved selecting original studies published in English, encompassing all studies conducted since the field's inception. Consequently, the final number of data-driven studies identified and chosen for this study amounts to 3967. Data-driven studies of CA-R provide a rigorous and reliable approach based on empirical evidence and systematic research methodologies. They offer objective analysis and enhance replicability and comparability of results. Restricting the study to English ensures wider accessibility and comprehension within the scientific community, facilitating global collaboration [16,26,30]. However, it is important to acknowledge the potential limitation of excluding non-English studies, which may introduce a language bias. The final data files were extracted and downloaded as CVS and BibTex formats.

### 2.4 Data analysis and visualization

The data analysis and visualization for this study utilized two widely used tools in bibliometric research: VOSviewer and Bibliometrix [31,32]. VOSviewer and Bibliometrix were used for both intellectual and conceptual structures.

#### 2.4.1 VOSviewer

VOSviewer is a versatile software tool designed to visualize and analyze bibliometric networks, enabling the exploration of co-authorship, co-citation, and keyword co-occurrence networks. It facilitates the identification of research trends, key topics, and influential authors or institutions within a specific field. By utilizing VOSviewer, this study was able to map and analyze the intellectual structure of CA-R.

#### 2.4.2 Bibliometrix

Bibliometrix, on the other hand, is an R package specifically developed for bibliometric analysis. It provides a range of functions and algorithms for calculating bibliometric indicators, such as citations, co-authorship networks, and keyword analysis. With the aid of Bibliometrix, this study conducted in-depth statistical analysis of bibliometric data, enabling the exploration of various aspects of CA-R.

#### 2.4.3 Intellectual structures

The bibliometric analysis in this study encompassed two main components: intellectual and conceptual structures [27]. The analysis of the intellectual structure involved identifying hotspots and differential production within CA-R, studying the growth of research and its impact through citation analysis from 1932 to 2023, and analyzing high-impact research to determine its research topics. This provided insights into the historical development and impact of CA-R.

#### 2.4.4 Conceptual structures

The study conducted an analysis of the conceptual structure to reveal the conceptual map, assess the growth and interconnection of topics, explore temporal growth patterns, and identify emerging themes within CA-R. This analysis shed light on the current state and future directions of CA-R, uncovering the relationships between various research topics. By combining these analyses, this study provides a comprehensive understanding of the historical development, current state, and future directions of CA-R, examining both quantitative aspects of research production and impact as well as qualitative aspects of research topics and their interrelationships [6,7,23,27].

In bibliometrics, centrality and density are measures used in thematic map analysis. Thematic maps visualize the intellectual structure of a research field by identifying clusters of related terms. Centrality measures the prominence of a term within a cluster or network, indicating its importance. Density measures the cohesion or interconnectedness of terms within a cluster, reflecting the level of relationships between them. These measures provide insights into influential topics, cohesive areas of research, and potential research gaps, aiding in the understanding of a field's knowledge landscape [26,31,32].

## 3 Results

### 3.1 Main information

Table 1 summarizes the CA-R-related documents spanning 91 years (1932–2023). It covers 3967 documents from 1065 journals, with a steady annual growth of 6.53 %. Key document characteristics include an average age of 11.2 years and 22.25 citations each. Over 12,000 authors contributed, with only 1.6 % single-authored, highlighting strong collaboration. Each document has an average of 5.25 authors, and nearly 30 % involve international collaboration.

**Table 1:** Main information.

Description	Results
Timespan	1932:2023
Sources (Journals)	1065
Documents (original articles)	3967
Annual Growth Rate %	6.53
Document Average Age (years)	11.2
Average citations per document	22.25
Keywords Plus (ID)	14586
Author's Keywords (DE)	8626
Authors	12028
Authors of single-authored docs	103
Authors collaboration	
Single-authored documents	116
Co-Authors per documents	5.25
International co-authorships %	29.35

### 3.2 Hotspots

**Fig. 2:**
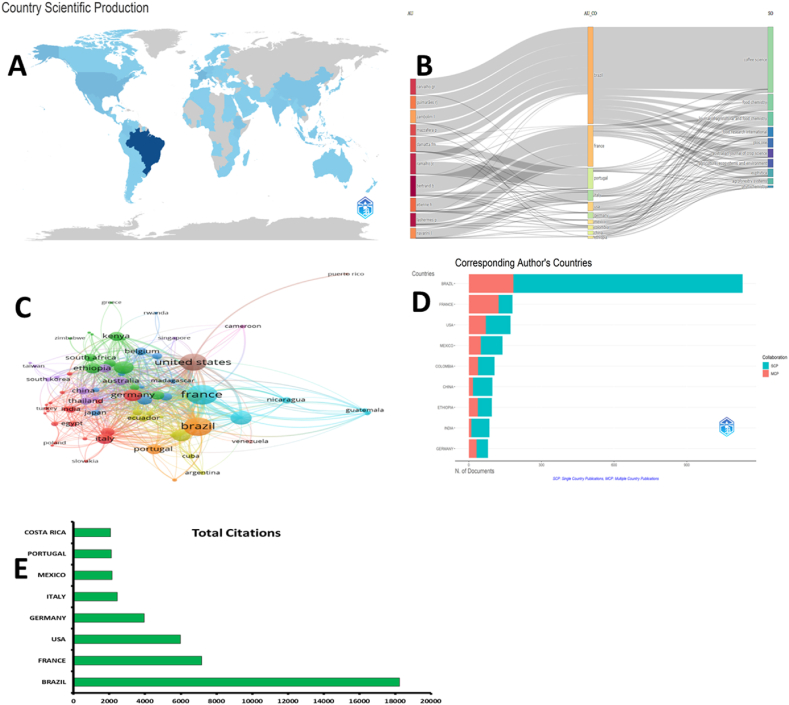
A: Global production in CA-R. Countries with a dark blue color are the most productive. Countries outside the blue category have not made any contributions to research in this particular area. This figure was generated using the Bibliometrix application and the BibTex data file. **B**: Sankey diagram. AU: authors; AU_CO: country of the authors; SO: sources. The thickness of the lines connecting authors from different countries represents the number of papers they have co-authored. The thickness of the lines connecting countries and sources represents the number of papers from each country that have been published in each source. Each rectangle represents an author, country, or source. The size of the rectangle represents the importance of nodes in the network. This figure was generated using the Bibliometrix application and the BibTex data file. **C:** Co-authorship networks among countries was analyzed using VOSviewer, where countries were represented as nodes and collaborations as links. Based on TLS values, France emerged as the leading collaborative country in CA-R. **D:** Single and multiple country publications. The orange color in the bars represents the multiple country publications**. E**: Highly cited countries.

The top fifteen countries in CA-R including Brazil, the United States, France, Mexico, Colombia, Ethiopia, Germany, Costa Rica, India, Italy, the United Kingdom, Portugal, China, Kenya, and Japan, have collectively produced 74.3 % of the total CA-R (Fig. 2A). Brazil leads the way with 1601 publications, accounting for 28.55 % of the total, followed by the United States with 398 publications (7.10 %) and France with 381 publications (6.80 %). With 34 publications on CA-R, Saudi Arabia represents 0.61 % of the CA-R. The most prolific scholars are Bertrand, B. with 70 publications, Lashermes, P. and Ramalho, J.C. with 48 publications. Etienne, H. has contributed 46 publications, and Carvalho, G.R. has 45 publications in the field. The most productive institutions in CA-R, based on the number of publications, are the Federal University of Lavras (Brazil) with 466 publications, the Federal University of Viçosa (Brazil) with 335 publications, CIRAD (French Agricultural Research Centre for International Development) with 265 publications, the Brazilian Agricultural Research Corporation - Embrapa with 198 publications, and Embrapa Coffee (Brazil) with 175 publications. The top journals include Coffee Science with 249 publications, Food Chemistry with 97 publications, Journal of Agricultural and Food Chemistry with 82 publications, Agroforestry Systems with 49 publications, Euphytica with 46 publications, and Phytochemistry with 45 publications.

In Fig. 2B, a Sankey Diagram (three field plot) was employed to visually represent the top ten prolific units in CA-R. The Sankey Diagram illustrates the flow of productivity based on three fields: authors, countries, and sources. Each unit, represented by a rectangle, reflects its level of productivity, with larger rectangles indicating higher productivity for a specific unit. The diagram analysis reveals that Bertrand, B. and Ramalho, J.C. secured the top spots in publishing output in the top ten journals associated with CA-R. These two authors demonstrated similar publication numbers, highlighting their significant contributions to the field. Regarding countries, France claimed the second position in productivity, indicating a strong research presence in CA-R. On the other hand, the United States fell to the fourth position, suggesting a relative decrease in research output compared with other countries. Regarding sources, the rankings experienced changes starting from the fourth position. However, it is worth noting that the three highest-published journals maintained their top positions, indicating their consistent status as the leading outlets for CA-R research.

### 3.3 Mapping of collaborative CA-R

The most collaborative countries can be identified based on the TLS (Total Links Strength) data obtained from VOSviewer mapping of country authorship (Fig. 2C). TLS is shown as node in this figure. Among the 75 countries mapped with a minimum of 5 documents, France emerges as the most collaborative country with a TLS of 489, followed closely by Brazil with a TLS of 430. The United States ranks third with a TLS of 371, while Costa Rica, the United Kingdom, Mexico, and Germany demonstrate significant collaboration with TLS values of 241, 199, 179, and 174, respectively.

Fig. 2D provides information on the single-country publications (SCP), multiple-country publications (MCP), and MCP ratio for different countries. The orange color in the bars represents the multiple country publication. Analyzing the SCP data, France has 57 SCPs out of 180 articles, accounting for 31.7 % of their total publications. The USA has 102 SCPs out of 172 articles, representing 59.3 % of their publications. Germany has 47 SCP out of 79 articles, making up 59.5 % of their output. Ethiopia has 58 SCPs out of 95 articles, accounting for 61.1 % of their publications. Colombia has 68 SCP out of 106 articles, representing 64.2 % of its output. Mexico has 90 SCPs out of 139 articles, making up 64.7 % of their publications. China has 80 SCPs out of 97 articles, accounting for a high percentage of 82.5 %. Brazil has the highest number of articles, with 1130, and a significant portion of 946 articles being SCP, representing 83.7 % of their publications. India has 75 SCP out of 85 articles, making up a substantial 88.2 % of their output. These figures illustrate the extent to which each country's research in CA is conducted within their own borders, indicating the level of self-reliance and internal collaboration within their respective research communities.

### 3.4 Annual production and impact

**Fig. 3:**
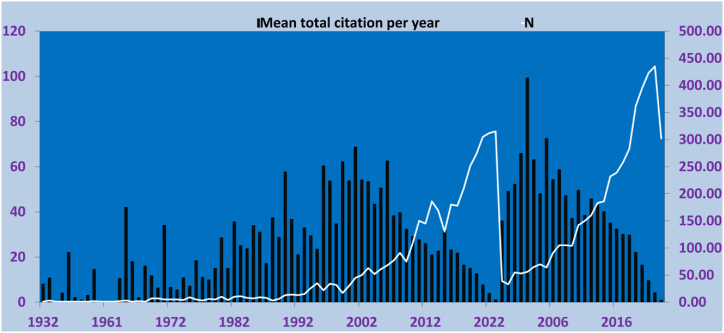
Annual production (lines) and mean total citation per year (Bars).

The number of CA-R-related documents varies across the years, with earlier years showing relatively low publication volumes and occasional spikes (Fig. 3). The 1980s witnessed an increase in article counts, while the 1990s saw a substantial rise in research activity, contributing approximately 7.46 % of the total articles. The early 2000s maintained a relatively high level of publication, followed by a consistently high research output period from 2007 to 2017. The 2010s accounted for around 45.33 % of the articles, highlighting a period of prolific research. In recent years, from 2021 to 2023, approximately 23.48 % of the articles were published, suggesting a noticeable but slightly lower level of research activity. These fluctuations in article counts over time reflect the dynamic nature of scientific research and offer valuable insights into the distribution of research output across different time periods. The mean number of citations per article varies across the years, ranging from 0 to 68.62. The highest mean number of citations per article is observed in 2001 with 68.62, followed by 1999 with 62.24 and 2003 with 53.35. The years with the lowest mean number of citations per article are 1938 with 0, 1959 with 0, and 1961 with 0.

### 3.5 Impactful CA-R

The most cited documents in CA-R cover various topics contributing to our understanding of this vital crop (Table 2). These include the fruit set of highland coffee and the role of pollinating bee diversity, shade management practices in coffee and cacao plantations, the green synthesis of silver nanoparticles using CA seed extract and its antibacterial activity, the impact of roasting on the formation of chlorogenic acid lactones in coffee, the categorization of seed storage behavior in coffee, the coffee rust crises in Colombia and Central America with proposed solutions, soil erosion prediction in central Kenyan highland conditions, the correlation between cup quality and chemical attributes of Brazilian coffee, the antioxidant activity of coffee brews and the effect of roasting, and the diversity of caffeine, trigonelline, chlorogenic acids, and sucrose in wild C. arabica and C. canephora accessions. These topics encompass various aspects of CA-R, pollination, cultivation practices, nanotechnology applications, chemical composition, roasting effects, disease management, environmental impact, and cup quality evaluation.

**Table 2:** Top-cited documents.

Rank	Title	Year	Source	Citation	Citation average
1st	(Fruit set of highland coffee increases with the diversity of pollinating bees	2003	Proceedings of the Royal Society B: Biological Sciences	569	27.10
2nd	Shade management in coffee and cacao plantations	1997	Agroforestry Systems	450	16.67
3rd	Green synthesis of silver nanoparticles using *Coffea arabica* seed extract and its antibacterial activity	2016	Materials Science and Engineering C	440	55.00
4th	Effect of roasting on the formation of chlorogenic acid lactones in coffee	2005	Journal of Agricultural and Food Chemistry	420	22.11
5th	An intermediate category of seed storage behaviour	1990	Journal of Experimental Botany,	397	11.68
6th	The coffee rust crises in Colombia and Central America (2008–2013): impacts, plausible causes and proposed solutions	2015	Food Security	361	40.11
7th	Soil erosion prediction using RUSLE for central Kenyan highland conditions. Agriculture	2003	Ecosystems and Environment	332	15.81
8th	Correlation between cup quality and chemical attributes of Brazilian coffee	2006	Food Chemistry	326	18.11
9th	Effect of roasting on the antioxidant activity of coffee brews	2002	Journal of Agricultural and Food Chemistry	325	14.77
10th	Caffeine, trigonelline, chlorogenic acids and sucrose diversity in wild *Coffea arabica* L. and C. canephora P. accessions	2001	Food Chemistry	298	12.96

### 3.6 Top-cited countries

The top-cited countries in CA-R are shown in Fig. 2E. Brazil has emerged as the most highly cited country, with 18,232 citations and an average of 16.10 citations per article. France has 7177 citations, with an average of 39.90 citations per article. The United States has 5974 citations, with an average of 34.70 per article. Germany ranks fourth, with 3960 citations and an average of 50.10 citations per article. Italy has 2444 citations, averaging 32.20 citations per article. Mexico, Portugal, and Costa Rica also showed a significant citation impact, with 2156 citations (average of 15.50 per article), 2123 citations (average of 31.70 per article), and 2066 citations (average of 48.00 per article), respectively. These top-cited countries demonstrate their research influence and prominence in the studied field.

### 3.7 Most frequent terms

**Fig. 4:**
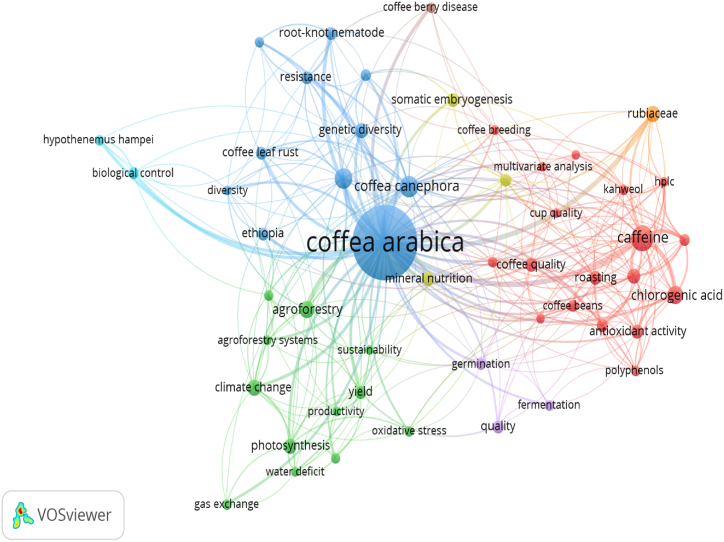
Network visualization generated using VOSviewer displaying the most frequent author's keywords in CA-R. The figure includes the top 50 terms out of a total of 8626 author's keywords. Each node represents the occurrence of a specific term, providing a visual representation of its relative frequency and interconnectedness. The figure provides insights into the important topics and relationships within the field, shedding light on key areas of interest and research focus related to CA.

Fig. 4, generated using network visualization in VOSviewer, illustrates the most frequently used authors' keywords based on the top 50 terms in CA-R. The total number of authors' keywords considered is 8626. In the visualization, nodes represent the occurrence of each term, allowing for a visual representation of the relative frequency and interconnectedness of these keywords. The network visualization provides insights into the important and recurring themes within the CA-R domain, highlighting the prominence and relationships between different author's keywords. The listed terms in Fig. 4 include *Coffea arabica* (2343), caffeine (145), chlorogenic acid (113), antioxidant (103), agroforestry (85), Hemileia vastatrix (83), genetic diversity (77), roasting (63), climate change (55), Rubiaceae (52), photosynthesis (50), coffee berry disease (47), polyphenols (47), Robusta coffee (40), coffee quality (39), mineral nutrition (39), Ethiopia (38), gene expression (38), root-knot nematode (38), somatic embryogenesis (38), coffee leaf rust (36), green coffee (36), resistance (35), yield (34), quality (32), trigonelline (31), biological control (30), coffee beans (30), germination (28), Hypothenemus hampei (28), biodiversity (27), kahweol (25), breeding (24), fermentation (24), oxidative stress (24), gas exchange (23), growth (23), coffee breeding (22), multivariate analysis (22), HPLC (21), sustainability (21), volatile compounds (21), water deficit (21), cup quality (20), diversity (20), productivity (20), cafestol (19), precision agriculture (19), remote sensing (19), and sensory analysis (19). These terms collectively represent the significant and recurring subjects within the CA-R. In the subsequent part, the author's keywords will be used to analyze the conceptual structure, thematic evolution, and emerging themes within the CA-R.

### 3.8 Conceptual evolution

**Fig. 5:**
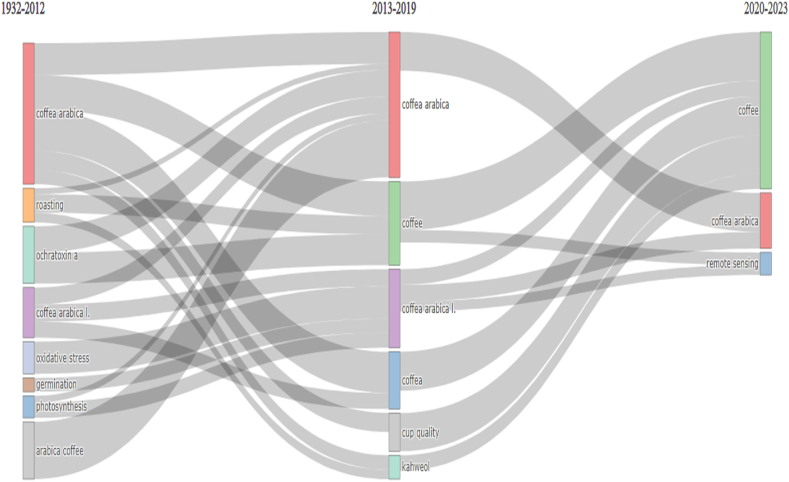
Thematic evolution of CA-R. 2012 and 2019 were crucial points for the transformation of the main topics. This figure was generated using the Bibliometrix application and the BibTex data file.

Fig. 5 depicts the thematic evolution of CA-R over time, particularly emphasizing the transformative points in 2012 and 2019. The figure was generated using the Bibliometrix application and the BibTex data file, enabling a comprehensive analysis of the thematic shifts within the CA-R. The thematic evolution of CA-R can be observed across different time periods. From 1932 to 2012, the main topics revolved around "arabica coffee," "*Coffea arabica* L.," "germination," "ochratoxin a," "oxidative stress," "photosynthesis," and "roasting." In the period of 2013–2019, there was a noticeable shift towards keywords such as "coffea," "coffee arabica," "cup quality," and "kahweol." This transition continued to evolve and gain prominence until 2019. From 2020 to 2023, new transitions occurred, including a further emphasis on "coffee" and the emergence of "remote sensing" as a relevant thematic area.

### 3.9 Mapping of CA-R

**Fig. 6:**
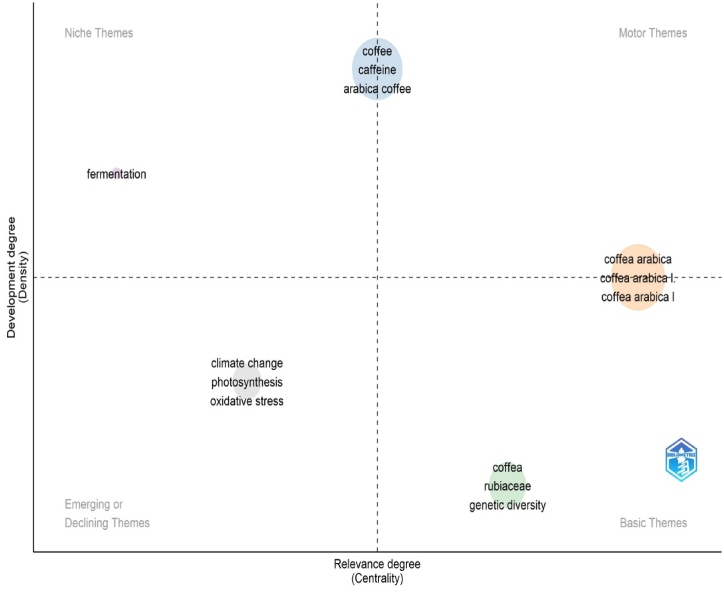
Thematic map of CA-R. Thematic maps are divided into four quadrants based on centrality and density, which represent the importance and development of research topics. This figure was generated using the Bibliometrix application and the BibTex data file.

Fig. 6 and Table 3 display the thematic map of CA-R, which was generated using the Bibliometrix application and the BibTex data file. The map is divided into four quadrants based on centrality and density, representing the importance and development of research topics. Five distinct clusters were identified on the thematic map. The first cluster, "Coffee," is characterized by a high centrality score of 0.056 and a dense distribution of research, with a density score of 4.664. This is classified as a theme that distinguishes between motor and niche themes. The second cluster, "Coffea," has a centrality score of 0.063 and a density score of 2.810, representing a foundational theme in the field. The "Fermentation" cluster has a lower centrality score of 0.006 but a high-density score of 4.167, indicating a niche theme within CA-R. The "*Coffea arabica* " cluster stands out with a centrality score of 0.090 and a density score of 3.925, classified as a theme that distinguishes between motor and basic themes. Finally, the "Climate change" cluster represents an emerging theme in CA-R, with a centrality score of 0.026 and a density score of 3.819. This thematic map analysis provides a comprehensive overview of the research landscape in CA-R, enabling researchers and stakeholders to identify key themes and their significance in Arabica coffee research.

**Table 3:** Terms and classifications of the themes in the thematic map of CA-R.

Cluster	Callon Centrality	Callon Density	Terms	Classification
Coffee	0.056	4.664	Coffee, caffeine, Arabica coffee, chlorogenic acid, roasting, coffee quality, antioxidant, green coffee, arabica, quality, trigonelline, coffee beans, kahweol, phenolic compounds, multivariate analysis, HPLC, robusta, volatile compounds, cup quality, cafestol, roasted coffee, robusta coffee, sensory analysis	Delineating between motor and niche themes
Coffea	0.063	2.810	Coffea, rubiaceae, genetic diversity, ethiopia, gene expression, somatic embryogenesis, germination, molecular markers, coffee berry disease	Basic theme
Fermentation	0.006	4.167	Fermentation	Niche theme
*Coffea arabica*	0.090	3.925	*Coffea arabica*, Coffea canephora, *Hemileia vastatrix*, agroforestry, mineral nutrition, root-knot nematode, coffee leaf rust, resistance, yield, biological control, *Hypothenemus hampei*, biodiversity, breeding, growth, coffee breeding, agroforestry systems, coffee berry borer, sustainability, water deficit, diversity, productivity, precision agriculture, remote sensing	Delineating between motor and basic themes
Climate change	0.026	3.819	climate change, photosynthesis, oxidative stress, gas exchange, coffee tree	Emerging

*Coffee refers to the beverage made from roasted coffee beans, while Coffea refers to the genus of plants that produce the coffee beans used for making coffee.

### 3.10 Emerging themes

**Fig. 7:**
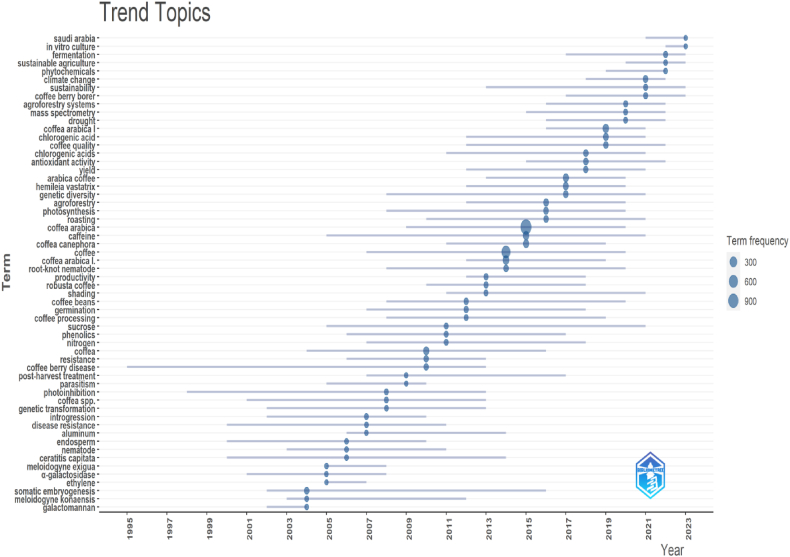
Trends in the CA-R. The graph depicts the research topic's time span, with horizontal lines indicating the duration and blue circles representing the frequency of the term. This figure was generated using Bibliometrix and BibTex data files.

The emerging themes in CA-R include In vitro culture, Saudi Arabia, sustainable agriculture, phytochemicals, climate change, coffee berry borer, and fermentation. Based on the provided data, the themes with the most extended shelf life in CA-R include coffee berry disease, photoinhibition, endosperm, *Ceratitis capitata*, and disease resistance. These terms have been consistently studied and mentioned over a significant period, ranging from 1995 to at least 2014. More details are shown in Fig. 7.

## 4 Discussion

The bibliometric assessment CA-R aims to comprehensively understand the research landscape, its impact, and contribute to informed decision-making, collaboration, and the advancement of Coffee Arabica cultivation and sustainability. Conducting this assessment fills a gap in dedicated bibliometric assessments in this field, providing insights into research trends, impact evaluation, and future research directions for CA-R.

Brazil is the dominant contributor to CA-R, accounting for approximately 28.55 % of total publications in the field. This leading position can be attributed to several factors. Brazil has witnessed significant growth in coffee production, with a 100 % increase in volume over the past three decades, making it responsible for 42 % of global coffee output in 2015. This demonstrates Brazil's strong presence and contribution to the coffee industry. Additionally, Brazil harvested 32 million bags of Arabica coffee and 11.2 million bags of Conilon species in 2015, highlighting its substantial production capacity and focus on Arabica coffee, a key aspect of CA-R. The country's extensive planted area of 2.3 million hectares and involvement of around 287,000 primarily small-scale farmers further indicate widespread participation in the industry. Moreover, Brazil's diverse geography, encompassing various climates, reliefs, altitudes, and latitudes, enables the production of a wide range of coffee types and qualities. This diversity further enhances Brazil's leading position in CA-R [33,34]. This diversity contributes to Brazil's leading position on the CA-R. These findings align with a previous bibliometric study that also recognized Brazil's leadership in this field [25].

Research collaboration mapping provides valuable insights into scientific research dynamics, facilitating informed decision-making, impactful collaboration, and knowledge creation. Visualizing collaboration networks helps identify trends, key players, interdisciplinary connections, and collaboration opportunities [16,26,30]. The TLS analysis reveals that multiple researchers contribute to each publication in CA-R, with around 29.35 % involving international collaborations [Table 1]. Previous studies have highlighted the landscape of coffee collaboration opportunities, guiding efforts to address research gaps and promote sustainable practices [35].

Notably, Bertrand, B., affiliated with the French Agricultural Research Centre for International Development, has made significant contributions to CA-R. His research covers diverse aspects, including genetic diversity, climatic influences, genetic introgression, biochemical composition, and chemical markers for variety discrimination and origin determination [2,9,10,[13], [14], [15], [16],[28], [29], [30]]. Bertrand's work advances understanding of CA genetics, environmental influences, and quality attributes, benefiting coffee industry stakeholders [2,13,14,[18], [19], [20], [21],[36], [37], [38]].

Research fields evolve due to a combination of internal and external drivers. Internal drivers include discoveries, methodologies, theoretical frameworks, research questions, and collaboration. External drivers encompass societal needs, funding, technology, politics, economics, and public interest. Understanding these drivers helps researchers anticipate trends and contribute to knowledge advancement [7,39]. The thematic evolution of CA-R shows a shift from fundamental aspects to broader exploration of coffee, including quality, health, and technology integration. This reflects the recognition of coffee's significance and consumer demand for specialty coffee. The inclusion of "remote sensing" indicates the adoption of advanced technologies for studying coffee. Overall, CA-R has become more interdisciplinary, encompassing various aspects beyond the Arabica plant itself [6,7,25,26,30].

Co-word analysis effectively maps the conceptual landscape of CA-R by examining keyword co-occurrence. It reveals core themes, emerging areas, and connections between them. This approach guides research, tracks field evolution, and facilitates interdisciplinary collaborations [7,26,30,40]. Thematic maps are divided into four quadrants based on centrality and density, which represent the importance and development of research topics. Callon Centrality and Callon Density are scientometric metrics used to assess the relevance and prominence of a term in a scientific field. Callon Centrality determines how central or essential a term is within a network of scientific publications, with higher values indicating greater centrality and influence. On the other hand, Callon Density measures the concentration or clustering of a term within the network, where higher values indicate a denser connection with different terms. These metrics provide valuable insights into the position and significance of a term within the scientific community, aiding researchers in understanding its relevance within the field [31]. Five clusters were extracted using co-word analysis (Fig. 6). This include “Coffee,” “Coffea,” “Fermentation,” “*Coffea arabica*,” and “Climate change.”

The study on climate change and citrus crops identified research areas, trends, and gaps. It focused on modeling, socio-political issues, and plant physiology. There was a shift towards studying plant physiology and stress responses, while noting the lack of investigations on combined stresses and predictive models for citrus production. Water use and irrigation alternatives were highlighted. By addressing these gaps, the scientific community can enhance understanding and develop strategies for climate change resilience in citrus cultivation. In comparison, the thematic map of CA-R would include research gaps such as limited focus on specific regions beyond Brazil, insufficient exploration of genetic diversity, and a need for climate change adaptation strategies. The gaps in post-harvest processing techniques and utilization of emerging technologies are also mentioned. On the other hand, the citrus study identified gaps in investigations on combined stresses and predictive models, as well as a need for water management solutions. These differences reflect the specific challenges and research priorities within each crop and highlight the areas where further scientific development is necessary.

The terms within the "Coffee" cluster cover various aspects of CA-R. For example, "caffeine" is a stimulant compound, while "Arabica coffee" specifically refers to the Coffea arabica species. "Chlorogenic acid" and "chlorogenic acids" are antioxidants in coffee, and "roasting" is the process of heating coffee beans for flavor development. Other terms such as "antioxidant activity," "coffee quality," "trigonelline," "polyphenols," and "phenolic compounds" explore coffee's chemical composition, sensory attributes, and potential health benefits. Classifying terms as motor and niche themes provides insights into the driving forces and specific subtopics within CA-R. The Coffee theme represents the main driving force, while terms like "cup quality," "sensory analysis," "volatile compounds," and "HPLC" focus on narrower areas of investigation [41]. Understanding these themes helps identify important areas of study and guide future research directions in CA-R.

Chlorogenic acid, trigonelline, kahweol, and cafestol are important secondary metabolites found in CA. Chlorogenic acid is a phenolic compound known for its antioxidant properties and potential health benefits. Trigonelline, an alkaloid, contributes to the aroma and flavor of roasted coffee and has been studied for its antioxidant and anti-inflammatory properties. Kahweol, a diterpene, provides the characteristic aroma and flavor of unfiltered coffee, and has been investigated for its potential anti-inflammatory and anti-cancer effects. Cafestol, another diterpene, is present in higher amounts in unfiltered coffee and contributes to its oily texture and aroma. While cafestol and kahweol have been studied for their potential impacts on cholesterol metabolism, filtered coffee generally contains lower levels of these compounds [[41], [42], [43]]. Understanding these secondary metabolites in CA enhances our knowledge of its chemical composition, sensory qualities, and potential health effects, contributing to both scientific research and the appreciation of coffee as a beverage.

Sensory analysis in CA-R involves the application of standardized sensory evaluation techniques to assess the sensory attributes and qualities specific to CA [18,22,44]. Trained sensory panelists evaluate coffee using established protocols, focusing on key sensory aspects, such as aroma, flavor, body, aftertaste, and overall quality. The evaluation includes assessing the fragrance and specific aroma notes, analyzing taste characteristics, including acidity and sweetness, examining the mouthfeel or body of the coffee, and considering the lingering aftertaste. Sensory analysis in CA-R serves various purposes, including quality control to maintain consistency and meet quality standards, developing sensory profiles that capture the unique characteristics of CA, guiding product development for new blends or processing techniques, understanding consumer preferences, and evaluating specialty coffees [44]. By leveraging sensory analysis, coffee professionals can gain valuable insights into the sensory profile of CA, ensuring a pleasant and satisfying coffee experience for consumers [18,22].

The "Coffea" theme terms include Coffea, Rubiaceae, genetic diversity, Ethiopia, gene expression, somatic embryogenesis, germination, molecular markers, and coffee berry disease. This theme primarily revolves around the basic aspects of Coffea, encompassing various topics such as genetic diversity, gene expression, somatic embryogenesis, germination, and the identification of molecular markers. These areas of study are crucial for understanding the fundamental characteristics and traits of coffee plants. Ethiopia is specifically mentioned, indicating its significance as a region known for its coffee production and genetic resources [19,37,42]. Including terms like "coffee berry disease" suggests a focus on the impact of diseases on Coffea plants and the efforts to mitigate their effects through genetic research and disease management strategies [45]. The theme highlights the importance of exploring the genetic diversity within Coffea species, which can provide insights into breeding programs, disease resistance, and the development of improved coffee varieties. Utilizing techniques such as gene expression analysis, somatic embryogenesis, and molecular markers demonstrates the application of molecular biology and biotechnology in advancing our understanding of Coffea plants [46]. This theme represents a foundational exploration of Coffea, encompassing genetic diversity, molecular aspects, and disease management. It sets the stage for further research and advancements in the cultivation, improvement, and protection of Coffea plants, ultimately contributing to the sustainability and quality of coffee production.

Coffee berry disease (CBD), caused by the fungus *Colletotrichum kahawae*, poses a significant threat to CA, the world's most popular coffee species [45]. It primarily affects coffee-producing regions in East Africa, such as Kenya, Ethiopia, and Tanzania, but can also impact crops in other parts of the world. CBD can infect coffee plants at any stage of growth and spreads through rain splash, wind, or contact with contaminated tools. The disease cycle involves the survival of the fungus in plant debris or fallen berries. CBD inflicts economic hardships on farmers, leading to substantial crop losses and reduced income. The disease can also decrease the quality of coffee beans, affecting taste, aroma, and overall sensory characteristics. To manage CBD, farmers and researchers employ various strategies. These include fungicide applications, breeding programs to develop resistant coffee varieties, implementing good agricultural practices, and exploring biological control methods [45,47,48]. Ongoing research focuses on improving diagnostics, understanding the disease's biology, and developing sustainable management strategies. Addressing CBD is crucial for the long-term sustainability of the coffee industry and the well-being of coffee farmers and communities worldwide.

Molecular markers have played a crucial role in coffee research, particularly in assessing genetic diversity. Limited information on the genetic diversity of commercial coffee cultivars in India over the past 90 years prompted a study using RAPD, ISSR, and SRAP markers. The analysis revealed higher genetic diversity with ISSR markers and identified two major clusters representing *C. arabica* and *C. canephora* [49]. Similarly, in Ethiopian coffee, RAPD markers highlighted distinct groups from different regions, suggesting diversity and showing little differentiation between cultivated coffee and wild coffee [10]. Another Kenyan study utilizing RAPD primers and microsatellites examined 24 coffee genotypes, uncovering clusters based on species and introgression [11,50,51]. These findings emphasize the value of molecular markers in assessing and understanding the genetic diversity of coffee, providing insights for breeding programs and genetic improvement efforts in the future.

The theme "Fermentation" in CA-R has a Callon Centrality of 0.006 and a Callon Density of 4.167, indicating it is a niche theme. Fermentation is a crucial process in coffee processing that significantly affects flavor and quality. It removes the mucilage layer, enhancing sweetness and clarity while eliminating undesirable flavors. Fermentation also contributes to the development of aroma precursors, resulting in distinct aroma and flavor profiles. Different fermentation methods produce a wide range of desirable flavor notes, allowing for customization. Fermentation ensures uniform drying and processing, consistent quality, and reduces the risk of defects. It can be tailored to enhance specific characteristics, aligning with preferences of different coffee origins and markets. Understanding the importance of fermentation enables producers to achieve diverse flavor profiles and cater to specific preferences [52,53].

In the thematic map of CA-R, the cluster "*Coffea arabica*" stands out, with a Callon Centrality of 0.090 and a Callon Density of 3.925. The classification of this cluster was delineated between motor and basic themes. In other words, it acts as a central and influential theme that connects and bridges various aspects of coffee agriculture research. This cluster encompasses various terms related to *Coffea arabica* and *C. canephora*, *Hemileia vastatrix* (coffee leaf rust), agroforestry, mineral nutrition, root-knot nematodes, resistance, yield, biological control, *Hypothenemus hampei* (coffee berry borer), biodiversity, breeding, growth, agroforestry systems, sustainability, water deficit, diversity, productivity, precision agriculture, and remote sensing.

Remote sensing has the potential to revolutionize the mapping of land cover and land use change (LCLUC) in coffee production areas. However, challenges such as the complexity of coffee production systems and limited access to satellite data have hindered progress Differentiating coffee from other vegetation using traditional methods is difficult due to heterogeneous landscapes and spectral similarities. Recent advancements in satellite technologies, including Synthetic Aperture Radar (SAR) sensors for cloudy regions and high-resolution optical sensors like Planet Labs' Dove satellites, along with the integration of moderate-resolution constellations like Landsat and Sentinel-2A/B, are addressing these challenges [54,55]. These advancements, coupled with the increasing demand for sustainable coffee production, provide an opportunity to improve remote sensing methods in coffee research, leading to better understanding and management of coffee production and its impact on land cover and land use [[54], [55], [56]].

In the thematic map of CA-R, a cluster titled "Climate Change" has a Callon Centrality of 0.026 and a Callon Density of 3.819. This cluster represents an emerging theme in coffee agriculture-research, highlighting the increasing importance of understanding the impact of climate change on coffee production. The terms associated with this cluster include climate change, photosynthesis, oxidative stress, gas exchange, and coffee tree. Climate change poses a significant challenge to coffee production and quality. The optimal growing conditions for Arabica coffee include specific temperature ranges, rainfall levels, and dry periods. However, coffee-producing regions are increasingly experiencing climatic conditions outside these optimal ranges, such as heat waves and droughts. For example, Central America is particularly vulnerable to climate change, with a projected reduction in suitable coffee-growing areas. In contrast, some regions in East Africa and Asia may see increased suitability for coffee production due to climate change, which could require land use changes and deforestation [12,13,16,18]. Climate change also affects coffee quality. The presence and concentration of primary and secondary metabolites influence the sensory attributes, shelf stability, and nutritional aspects of coffee. A balanced cup with specific levels of acidity, body, flavor, and aftertaste attributes often defines high-quality coffee. The composition of secondary metabolites, including caffeine, trigonelline, chlorogenic acids, and volatile compounds, contribute to the sensory profile and aroma perception of coffee. Climate change can alter the concentrations of these compounds, affecting the overall quality and flavor of coffee. Coffee quality is influenced at each production stage, from farming to postharvest processing and preparation. Factors such as genetics, climate, management conditions, and harvest practices affect farm coffee quality. Post-harvest processes, storage, and preparation methods also play a role. Climate change can reduce the acidity and flavor of coffee in low-altitude regions. At the same time, supplemental irrigation has been shown to improve coffee quality in areas with low and erratic rainfall. Management practices such as fruit thinning and fungicide application can also affect the volatile compound composition and perceived quality of the prepared coffee. Ultimately, the sensory and secondary metabolite profiles that determine coffee quality have economic implications as they influence consumer purchasing decisions [12,13,[16], [17], [18],^22^,^26^,^48^]. Understanding the impact of climate change on coffee production and quality is crucial for the coffee industry to adapt to and mitigate potential negative effects. A study on climate change and citrus crops identified three main research areas: modeling, socio-political issues, and plant physiology. The main topics in climate change research in citrus were “agricultural production,” “irrigation,” “dicolyledon,” “water resources,” “human activities,” “vegetable,” “agricultural management,” “plantation,” “climate change,” “irrigation,” “carbon sequestration,” and “physiology.” There has been a shift in research focus from modeling and risk analysis to plant physiology and stress studies. The positive impacts of climate change on certain citrus crops, indicating the need for comprehensive studies considering different climate scenarios [23]. Research gaps include the lack of investigations on combined stresses and predictive models for citrus production under various climate conditions. There is also growing interest in studying water use and irrigation alternatives to address water scarcity and improve production system resilience. Overall, further research is needed to understand the interactions between climate change and coffee production and develop strategies for adaptation and sustainability.

Sustainability is a critical and emerging theme in CA-R, encompassing environmental, social, and economic considerations. Researchers focus on mitigating the environmental impact of coffee cultivation through agroforestry, soil conservation, water management, and reduced chemical inputs. They also study carbon footprint and climate change adaptation. Social sustainability involves fair trade, equitable market access, labor conditions, gender equity, and improving livelihoods for farmers. Inclusive and participatory approaches are promoted. Economic sustainability includes market dynamics, value chain analysis, and economic viability. Factors like price fluctuations, market demand, certification schemes, and diversification strategies are studied to develop sustainable business models and enhance profitability [12,35,57].

The emergence of "Saudi Arabia" as a significant player in coffee agriculture research (CA-R) is due to several factors. The Saudi Arabian government has actively supported coffee research by providing funding, infrastructure development, and establishing research centers. Non-governmental organizations (NGOs) have also played a role in supporting research initiatives [1,[3], [4], [5],^9^,^10^,^58^]. Jazan University, particularly its Environmental Research Center, has shown a keen interest in coffee research. The geographical landscape of Saudi Arabia, including its mountainous regions, provides suitable environments for coffee cultivation and field studies. Coffee holds significant cultural importance in Saudi Arabia, being deeply ingrained in customs, traditions, and art forms. In recognition of Saudi Arabia's coffee heritage and research advancements, 2022 was designated as "The Year of Saudi Coffee [[59], [60], [61]].

## 5 Limitations

The study has limitations such as relying solely on the Scopus database, which may have missed research from other sectors, and having a language bias by focusing only on English-language papers. Also, using only one data source might have excluded valuable information from other sources. To enhance the traditional analytical method, different types of literature reviews could be incorporated. It is crucial to periodically update the study to include new articles in databases, and future research could utilize multiple databases for a more comprehensive analysis. Lastly, the study proposes improving the search method by adopting a multi-method approach or refining the search strategy to gain a better understanding of research trends in the sector.

## 6 Conclusions

CA-R has experienced steady growth and strong international collaboration, with Brazil leading in productivity and citations. Key studies have focused on pollination, shade management, nanotechnology applications, roasting effects, disease management, and environmental impacts. The thematic evolution of CA-R reveals shifts in research focus over time, with recent emphasis on cup quality, coffee arabica, and emerging themes like remote sensing. The analysis highlights five clusters: coffee, coffea, fermentation, coffea arabica, and climate change. Emerging themes include *in vitro* culture, Saudi Arabia, sustainable agriculture, phytochemicals, coffee berry borer, and fermentation. These findings contribute to improving cultivation practices, cup quality, and addressing challenges in the coffee industry.

Research gaps in CA-R include limited exploration of coffee-growing regions beyond Brazil, insufficient investigation of Coffee Arabica's genetic diversity for breeding and disease resistance, and a need for strategies to adapt to climate change. Further research is needed on post-harvest processing techniques and emerging technologies in the coffee industry. Addressing these gaps would lead to a more comprehensive understanding of CA and provide solutions to industry challenges. Future perspectives in CA-R involve developing climate-resilient varieties, sustainable farming practices, improving flavor and quality, understanding consumer preferences, adopting digital technologies, addressing social and economic sustainability, and researching health benefits. Collaboration is crucial for achieving sustainable and innovative coffee production. Recommendations include fostering international collaboration, supporting emerging research themes, strengthening key topics, promoting research funding and scholarships, enhancing knowledge exchange platforms, and promoting sustainable coffee practices. These recommendations aim to advance CA-R and contribute to the sustainable growth of the coffee industry.

## Ethics approval and consent to participate

There is no form of human subject involved in this manuscript; therefore, ethics approval is not required.

## Consent for publication

Not applicable.

## Funding

The authors gratefully acknowledge the funding of the Deanship of Graduate Studies and Scientific Research, 10.13039/100009388Jazan University, Saudi Arabia, through Project Number: RG24-M024.

## Availability of data and material

The datasets used and/or analyzed during the current study available from the corresponding author on reasonable request.

## CRediT authorship contribution statement

**Siddig Ibrahim Abdelwahab:** Writing – review & editing, Writing – original draft, Visualization, Validation, Supervision, Software, Resources, Project administration, Methodology, Investigation, Funding acquisition, Formal analysis, Data curation, Conceptualization. **Manal Mohamed Elhassan Taha:** Writing – review & editing, Writing – original draft, Visualization, Validation, Supervision, Software, Funding acquisition, Formal analysis, Data curation, Conceptualization. **Ahmed Ali Jerah:** Writing – review & editing, Writing – original draft, Data curation, Conceptualization. **Ieman A. Aljahdali:** Validation, Supervision, Software, Resources, Project administration, Methodology, Investigation, Funding acquisition, Formal analysis, Data curation, Conceptualization. **Bassem Oraibi:** Writing – review & editing, Writing – original draft, Project administration, Conceptualization. **Hassan Ahmad Alfaifi:** Writing – review & editing, Writing – original draft, Visualization, Data curation, Conceptualization. **Saleh M. Abdullah:** Writing – review & editing, Writing – original draft, Data curation, Conceptualization. **Amal Hamdan Alzahrani:** Writing – review & editing, Writing – original draft, Formal analysis, Conceptualization. **Omar Oraibi:** Writing – review & editing, Writing – original draft, Visualization. **Yasir Babiker:** Writing – review & editing, Data curation, Conceptualization. **Abdullah Farasani:** Writing – review & editing, Writing – original draft, Data curation, Conceptualization.

## Declaration of competing interest

The authors declare that they have no known competing financial interests or personal relationships that could have appeared to influence the work reported in this paper.
